# Pathological changes in various organs in HLA-B*57:01 transgenic mice with abacavir-induced skin eruption

**DOI:** 10.1007/s43188-023-00220-1

**Published:** 2024-01-06

**Authors:** Akira Kazaoka, Kazuyoshi Kumagai, Junya Matsushita, Tetsuo Aida, Saki Kuwahara, Shigeki Aoki, Kousei Ito

**Affiliations:** 1 Laboratory of Biopharmaceutics, Graduate School of Pharmaceutical Sciences, Chiba University, 1-8-1 Inohana, Chuo-ku, Chiba-City, Chiba 260-8675 Japan; 2 Medicinal Safety Research Laboratories, Daiichi Sankyo Co., Ltd, Tokyo, Japan

**Keywords:** HLA, Idiosyncratic adverse drug reaction, Abacavir hypersensitivity, Drug eruption

## Abstract

Several patients with cutaneous adverse drug reactions exhibit extracutaneous organ damages, and it becomes severe in a few patients resulting in death due to multiorgan failure. Understanding the sequential changes in various organs in patients with cutaneous eruption following drug administration will help understand disease onset and progression, aiding the development of prevention strategies and interventions. Therefore, we aimed to understand the effects of abacavir (ABC) on various organs in patients with ABC-induced eruptions by evaluating its effects in a mouse model. We found pathological changes in various organs of HLA-B*57:01 transgenic mice (B*57:01-Tg) following oral administration of ABC (20 mg/body/day). B*57:01-Tg exhibited a significant body weight decrease from day 1 of ABC administration, and reddening of the auricle was observed from day 5, and approximately 2/3 mice died by day 7. Histopathological examination revealed severe thymic atrophy after day 3, infiltration of inflammatory cells, predominantly lymphocytes with neutrophils, not only in the skin but also in the liver, kidney, and lung after day 5, and an increased number of lymphocytes with enlarged nuclei and granulocytic hematopoiesis were observed in the spleen after day 5. Blood chemistry revealed that albumin/globulin ratio was below 1.0 on day 5, reflecting a systemic inflammatory response, and the aspartate aminotransferase concentration rose to 193 ± 93.0 U/L on day 7, suggesting that cell damage may have occurred in various organs including liver accompanying inflammatory cell infiltration. These examinations of a mouse model of ABC-induced skin eruption show that disorders in various organs other than the skin should be considered and provide insights into the unexpected early systemic responses dependent on HLA-B*57:01.

**Supplementary Information:**

The online version contains supplementary material available at 10.1007/s43188-023-00220-1.

## Introduction

In several patients with cutaneous adverse drug reactions, organs other than the skin are damaged or inflamed, and some reactions cause severe organ damage leading to organ failure complications or even death [1, 2]. For example, patients with cutaneous adverse reactions induced by abacavir (ABC; a nucleoside analog, an anti-HIV drug), concomitantly develop fever, malaise, gastrointestinal symptoms, lymph node enlargement, and other organ disorders, such as liver damage [3, 4]. A total of 49% of the patients with a diagnosis of ABC hypersensitivity (AHS) reactions had the involvement of 3 or 4 body systems [3]. Some patients present with acute respiratory distress syndrome that requires mechanical ventilation or liver injury with blood aspartate aminotransferase (AST) levels > 1000 U/L and alanine aminotransferase (ALT) levels > 500 U/L in addition to the skin eruption [5, 6]. However, the characteristics of other organ disorders in patients with drug eruptions have not been elucidated because of the diverse combinations of organ disorders and lack of conducted or reported biopsies for minor organ damage. For example, there are few reports on the histopathology of patients with AHS, and the existing reports are limited to MRI or X-ray photographs [5, 6]. Therefore, understanding the sequential changes in various organs in patients with drug eruption will provide information on the disease onset and progression, aiding the development of prevention strategies and interventions.

Clinically, once an inflammatory reaction is triggered in a particular organ, the identification of disorders in other organs is challenging, and this difficulty is not exclusive to drug eruptions. Animal models with specific organ inflammation have been analyzed to address this issue. For example, the effects on various organs during the development of arthritis have been indicated using adjuvant-induced arthritic rats and collagen-induced arthritic mice [7–9]. These models have contributed to the prediction of liver function changes in patients with arthritis. Therefore, animal models of drug eruption are expected to contribute to the prediction of changes in various organs in patients with drug eruptions.

Some cutaneous adverse drug reactions are strongly associated with human leukocyte antigen (HLA) alleles. AHS is known to occur in some HLA-B*57:01 carriers (positive predictive value: 47.9%; negative predictive value: 100%) and is characterized by maculopapular eruptions [4, 10–12]. The median time to onset of response is 8 days after starting oral ABC administration [4]. The reproduction of skin eruptions and immune activation caused by ABC was performed using HLA-B*57:01 transgenic mice [13–15]. For example, HLA-B*57:01 transgenic mice develop skin eruptions, infiltration of CD8^+^ T cells into the skin tissue, and increased serum C–C motif chemokine (CCL) 17 levels after oral administration of ABC for 1 week [15, 16]. In our HLA-B*57:01 transgenic mice, we observed enlarged cervical, brachial, axillary, inguinal, and mesenteric lymph nodes and spleens, and an increased percentage of effector memory CD8^+^ T cells in the spleen, owing to a systemic immunological effect [15].

Therefore, investigating the effects of ABC administration on HLA-B*57:01 transgenic mice can offer insights into predicting the changes in various organs in patients with AHS. This study reveals the pathological changes in various organs of HLA-B*57:01 transgenic mice following the administration of ABC.

## Materials and methods

### Materials

ABC sulfate was purchased from Carbosynth Ltd. (Compton, Berkshire, UK). A rat anti-mouse CD4 monoclonal antibody (mAb) (clone GK1.5) was purchased from BioLegend (San Diego, CA, USA). Rat anti-CD8a mAb (clone YTS169.4) and Alexa Fluor 488-conjugated secondary antibody were purchased from Abcam (Cambridge, UK). Rabbit anti-cytokeratin 16 mAb (clone 8L6R4), FITC-conjugated secondary antibody, and Hoechst 33342 were purchased from Thermo Fisher Scientific (Waltham, MA, USA).

### Animals

**Fig. 1:**
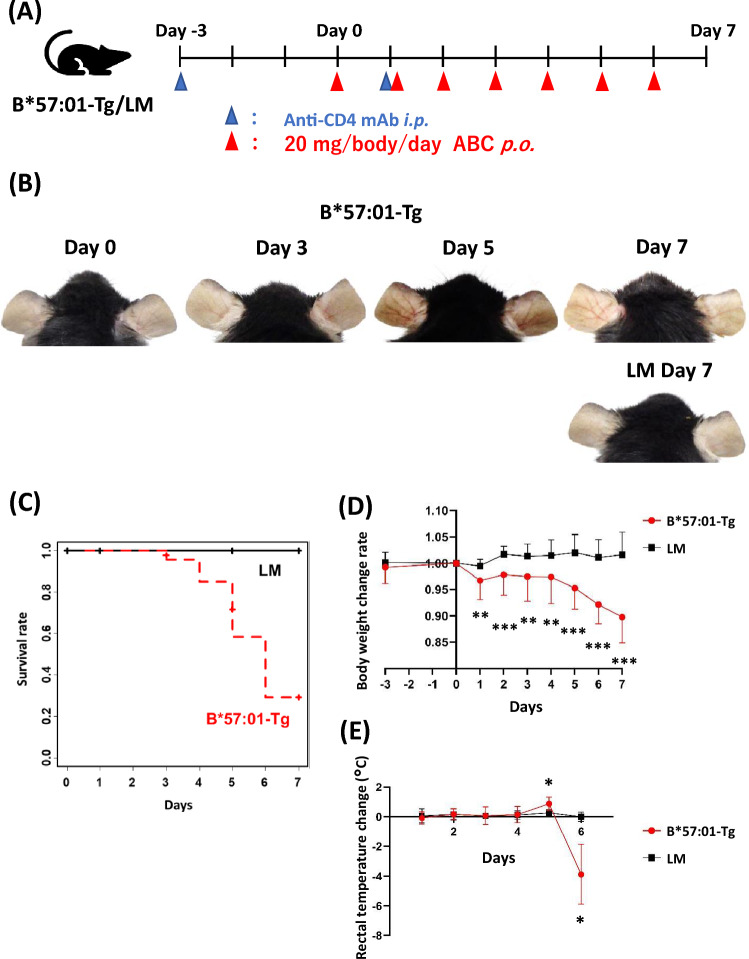
Follow-up observation in abacavir (ABC)-administered mice. Treatment schedule (**a**), photo of auricular skin (**b**), Kaplan–Meier survival curve (**c**), body weight change rate from baseline (**d**), and rectal temperature change from baseline (**e**) in B*57:01-Tg and their littermates (LM) with oral administration of ABC. Photo images are representative of 3–10 independent experiments. Data are expressed as the mean ± S.D. (n = 4–29/group). There were significant differences (*p < 0.05, **p < 0.01, ***p < 0.001) between B*57:01-Tg and LM (*t*-tests)

Previously, we established an HLA-B*57:01 transgenic mouse model in which skin eruption was induced by the oral administration of ABC [15]. Briefly, in HLA-B*57:01 transgenic mice, PD-1 was genetically knocked out, and CD4^+^ T cells were depleted using anti-CD4 mAb. In this study, we used HLA-B*57:01 transgenic mice subjected to these treatments (B*57:01-Tg) and their non-transgenic littermates (LM; mice with PD-1-knockout and CD4^+^ T-cell depletion). LM was used as a negative control [15]. Male mice (9–13 weeks old) were treated with intraperitoneal injections of 0.25 mg/body anti-CD4 mAb on day -3 and day 1 for CD4^+^ T-cell depletion, and 20 mg/body/day ABC oral administration suspended in 1% carboxymethyl cellulose aqueous solution on days 0–6 (day 0 as the starting date of ABC administration, Fig. 1A). The doses and treatment duration of ABC given by oral administration were established with reference to a previous paper [15]. Body weight was measured immediately before the administration of mAb/ABC, and rectal temperature was measured. The animals were treated in accordance with the guidelines issued by the National Institutes of Health. All the procedures were approved by the Animal Care Committee of Chiba University.

### Histopathological assessment

Tissue sampling was conducted on day 0 (before administration of ABC), 1, 3, 5, or 7, respectively. The brain, liver, heart, lung, kidney, thymus, spleen, cervical and mesenteric lymph nodes, and skin (auricle and back) were fixed in 10% neutral buffered formalin (Nacarai Tesque; Kyoto, Japan). These tissues were sectioned, embedded in paraffin (Sakura Finetek; Tokyo, Japan), and stained with hematoxylin (Muto Pure Chemicals; Tokyo, Japan) and eosin (Sakura Finetek) using a routine procedure. All histopathological evaluations were conducted by a board-certified veterinary pathologist.

### Immunohistochemistry

The auricles, livers, and kidneys were collected, embedded in Tissue-Tek® O.C.T. Compound (Sakura Finetek), and sliced into 5 μm thick sections using a cryostat. Cryosections were fixed in acetone and incubated with rat anti-CD8a mAb followed by incubation with an antibody to rat IgG conjugated to Alexa Fluor 488 and Hoechst 33342 [15]. Cryosections were fixed in 4% paraformaldehyde and permeabilized in 0.2% Triton X-100, and incubated with rabbit anti-cytokeratin 16 (K16) Ab followed by incubation with an antibody to rabbit IgG conjugated to FITC and Hoechst 33342.

### Measurement of serum biochemical parameters and cytokine and chemokine levels

Serum biochemical analysis was performed using TBA-2000FR (Toshiba Medical Systems Corporation; Ohtawara, Japan) for the following parameters: AST, ALT, alkaline phosphatase (ALP), creatine kinase (CK), blood urea nitrogen (BUN), total protein (TP), albumin (ALB), globulin (GLB), albumin/globulin ratio (A/G). The serum levels of mouse cytokines and chemokines were measured by Milliplex MAP Mouse Cytokine/Chemokine Magnetic Bead Panel (Merck Millipore; Darmstadt, Germany) in a BioPlex® system (Bio-Rad Laboratories; Hercules, CA, USA). The assay was performed according to the manufacturer’s protocols. Serum CCL17 levels were measured using a Mouse CCL17/TARC DuoSet enzyme-linked immunosorbent assay kit (R&D Systems, Inc.; Minneapolis, MN, USA) as previously described [16].

### Statistical analysis

All data are presented as mean ± S.D. Significance was determined using Student’s *t*-test for comparisons between two groups or Dunnett’s test for multiple comparisons following one-way analysis of variance. Statistical significance was set at *p* < 0.05. Statistical analyses were performed using the GraphPad Prism 8 software (GraphPad Software; La Jolla, CA, USA). Survival curves were plotted using the Kaplan–Meier method with R i386 4.1.2. software (R Foundation; Vienna, Austria). All experiments have been replicated at least three mice/group and are reproducible.

## Results

### Follow-up observation in ABC-administered mice

In B*57:01-Tg, daily ABC administration induced skin redness in the auricle from day 5 (Fig. 1B). Several B*57:01-Tg were found dead, most of them between days 5 and 7, with a survival rate of 28% on day 7 (Fig. 1C). B*57:01-Tg showed weight loss from the day after the start of treatment, with a trend toward further weight loss from day 5 onward (Fig. 1D). Furthermore, B*57:01-Tg exhibited a transient increase in body temperature on day 5 and a marked decrease on day 6 (Fig. 1E). In contrast, LM did not show any auricular redness, weight loss, body temperature changes, or death after ABC administration (Fig. 1).

### Histopathological examination of various organs

**Fig. 2:**
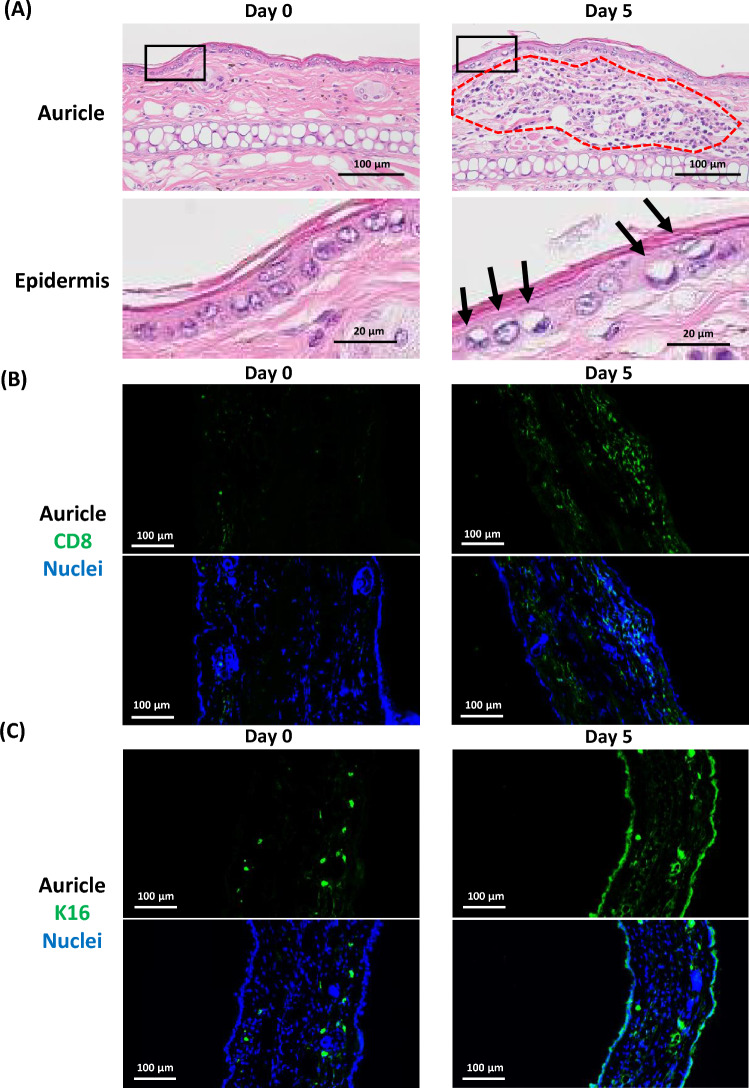
Histopathological examination of auricular skin. Representative images of the sections of auricular skin stained with hematoxylin and eosin (**a**), and the sections of auricular skin stained with anti-CD8 antibody (green) and Hoechst 33342 (blue; nucleus staining) (**b**), and the sections of auricular skin stained with anti-cytokeratin 16 antibody (green) and Hoechst 33342 (blue) in B*57:01-Tg with oral administration of abacavir (**c**). Images are representative of 3–5 independent experiments. Infiltration of lymphocytes with neutrophils (red dotted circle) and enlarged nucleus (arrows) are shown in the images

Histopathological examination of B*57:01-Tg and LM was performed at 0, 1, 3, 5, and 7 days after ABC administration. In the B*57:01-Tg auricular skin, the infiltration of lymphocytes and neutrophils was observed in the dermis and subcutaneous tissue after day 5 (Fig. 2A, Supplementary Fig. 1A, Table 1). Some of the infiltrated cells were CD8-positive in B*57:01-Tg (Fig. 2B, Supplementary Fig. 1B). The nuclei of the epidermal epithelium tended to be enlarged on day 5 compared with those on the days before, suggesting possible changes in the epidermal epithelium (Fig. 2A, Supplementary Fig. 1A). K16 is an intermediate filament protein in the skin that is an inducible keratin upon stress or inflammation and characteristic of hyperproliferative keratinocytes [17]. Immunostaining revealed increased K16 expression in the epidermis of B*57:01-Tg after day 5 compared with that on the days before (Fig. 2C, Supplementary Fig. 1C). These findings were not observed in the auricular skin of the LM, even on day 7 (Supplementary Fig. 1, Table 1).

**Table 1:** Histopathological findings considered to be related with abacavir administration

Organ	Histopathological finding	Mouse	B*57:01-Tg					Littermates (non-Tg)	
		Abacavir (Day)	0	1	3	5	7	0	7
Liver	(Number examined)		(3)	(3)	(3)	(5)	(4)	(3)	(3)
	Inflammatory cell infiltrate, lymphocyte and neutrophil		0	0	0	5	4	0	1
Lung	(Number examined)		(3)	(3)	(3)	(5)	(4)	(3)	(3)
	Inflammatory cell infiltrate, lymphocyte and neutrophil		0	1	1	4	2	0	0
Kidney	(Number examined)		(3)	(3)	(3)	(5)	(4)	(3)	(3)
	Inflammatory cell infiltrate, lymphocyte and neutrophil		0	0	0	3	1	0	0
Thymus	(Number examined)		(3)	(3)	(3)	(5)	(3)	(3)	(3)
	Single cell death of lymphocyte in cortex (slight)		0	0	2	0	0	0	3
	Single cell death of lymphocyte in cortex (severe)		0	0	1	5	3	0	0
Spleen	(Number examined)		(3)	(3)	(3)	(5)	(4)	(3)	(3)
	Increased number of lymphocyte with enlarged nucleus		0	0	1	5	3	0	0
	Granulocytic hematopoiesis in red pulp		0	0	1	5	4	0	0
Mesentery lymph node	(Number examined)		(3)	(3)	(3)	(5)	(4)	(3)	(3)
	Increased number of lymphocyte with enlarged nucleus in paracortex/medullary cord		0	1	5	3	0	0	0
	Single cell death of lymphocyte in paracortex/medullary cord		0	0	0	5	1	0	0
	Inflammatory cell infiltrate in medullary sinus, neutrophil								
Cervical lymph node	(Number examined)		(3)	(3)	(3)	(4)	(3)	(3)	(3)
	Increased number of lymphocyte with enlarged nucleus in paracortex/medullary cord		0	1	4	2	0	0	0
	Single cell death of lymphocyte in paracortex/medullary cord		0	0	0	4	2	0	0
	Inflammatory cell infiltrate in medullary sinus, neutrophil		0	0	0	4	2	0	0
Skin (ear)	(Number examined)		(3)	(3)	(3)	(5)	(4)	(3)	(3)
	Inflammatory cell infiltrate, lymphocyte and neutrophil		0	0	0	4	4	1	1

No finding considered to be related with abacavir was observed in the heart, cerebrum, or cerebellum of any groups

**Fig. 3:**
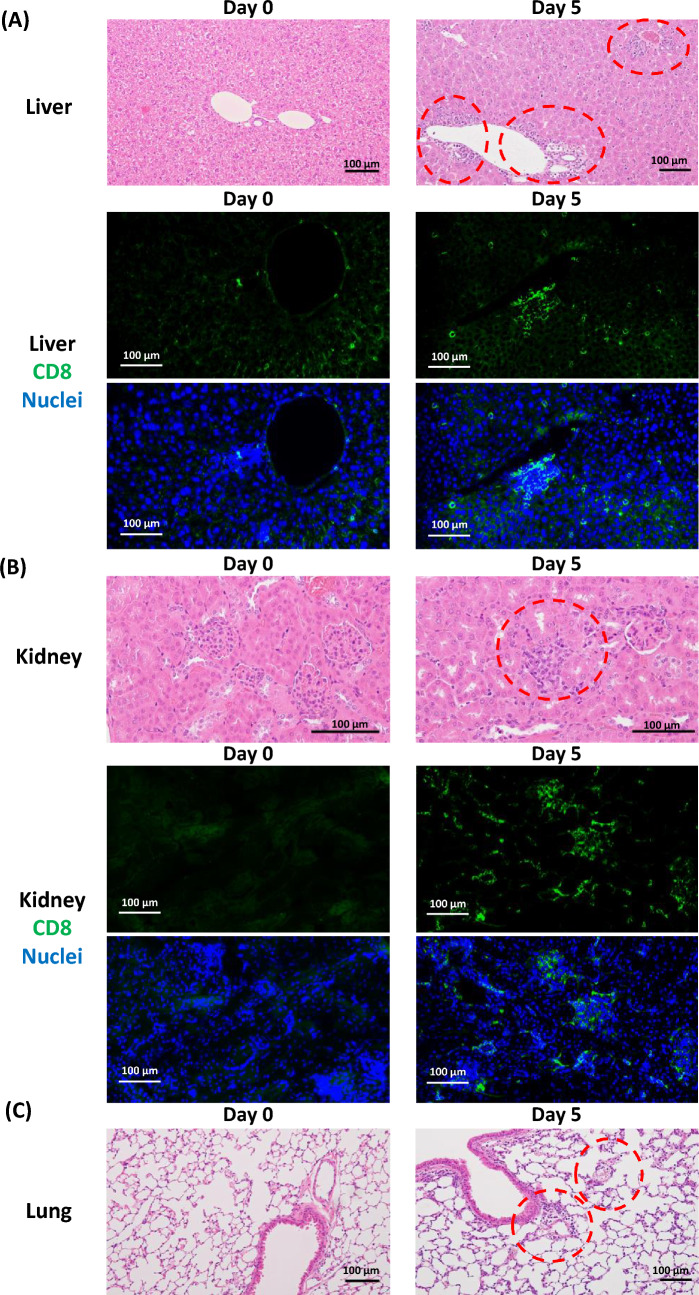
Histopathological examination of liver, kidney, and lung. Representative images of the sections of liver (**a**), kidney (**b**), and lung (**c**) stained with hematoxylin and eosin and with anti-CD8 antibody (green) and Hoechst 33342 (blue; nuclear staining) in B*57:01-Tg with oral administration of abacavir. Images are representative of 3–5 independent experiments. Infiltration of lymphocytes with neutrophils (red dotted circle) are shown in the images

In B*57:01-Tg, lymphocyte and neutrophil infiltration was observed in the liver, kidney, and lung after day 5 (Fig. 3, Supplementary Fig. 2, Table 1). In the liver, the foci of inflammatory cells tended to be distributed in the central or peripheral areas of lobules (Fig. 3A, Supplementary Fig. 2A, Table 1). The inflammatory cell foci were also observed in the interstitial tissues of the kidney and lung (Fig. 3B, C, Supplementary Fig. 2B, C, Table 1). Immunostaining confirmed that some of these were CD8-positive (Fig. 3A, B, Supplementary Fig. 2A, B). These findings were not observed in the liver, kidney, or lung of LM on day 7 (Supplementary Fig. 2, Table 1).

**Fig. 4:**
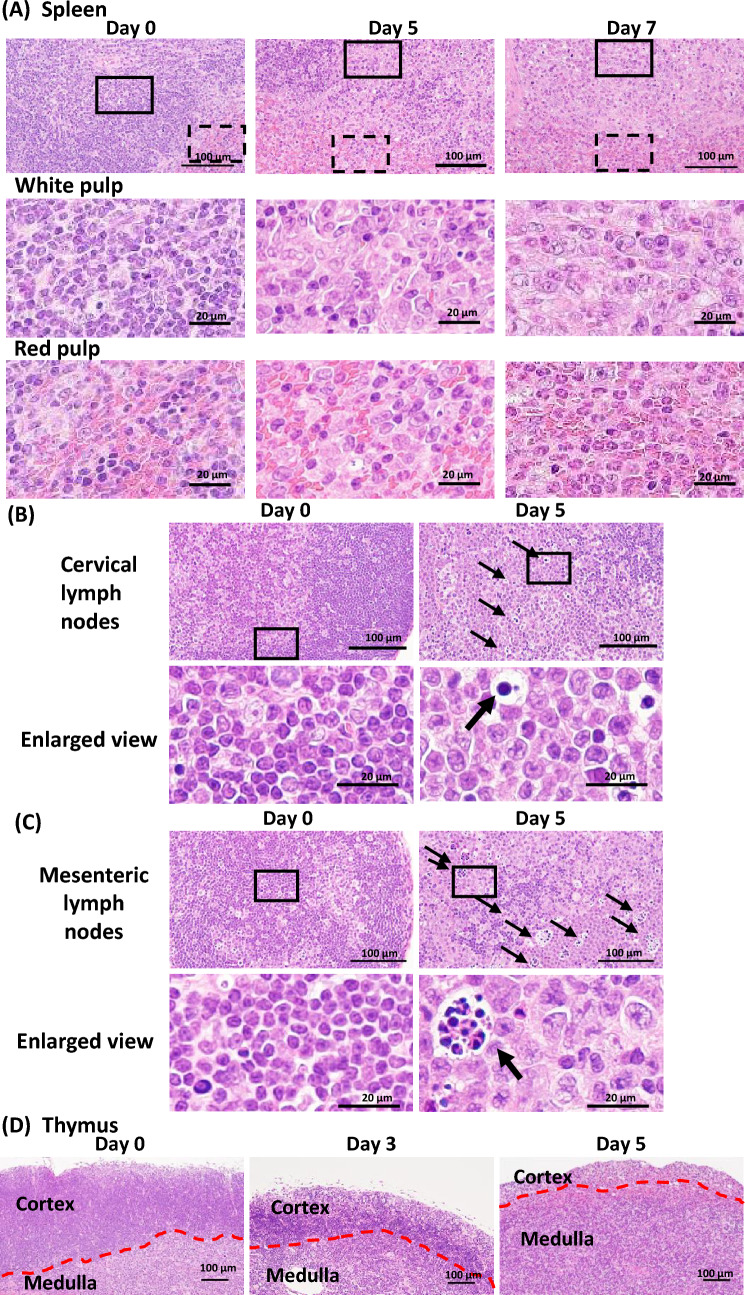
Histopathological examination of lymphoid tissues. Representative images of the sections of spleen (**a**), cervical lymph node (**b**), mesenteric lymph node (**c**), and thymus (**d**) stained with hematoxylin and eosin in B*57:01-Tg with oral administration of abacavir. Images are representative of 3–5 independent experiments. Single cell death of lymphocytes (arrows) and boundary between medulla and cortex (red dotted lines) are shown in the images

The lymphoid tissue was also affected by ABC administration in B*57:01-Tg. In the spleen, an increased number of lymphocytes with enlarged nuclei in the white pulp and increased granulocytic hematopoiesis in the red pulp were observed after day 5 (Fig. 4A, Supplementary Fig. 3A, Table 1). The increase in granulopoiesis was more remarkable on day 7 (Fig. 4A, Supplementary Fig. 3A, Table 1). In the cervical and mesenteric lymph nodes, an increased number of lymphocytes with enlarged nuclei were observed in the paracortex and the medullary cord after day 5 (Fig. 4B, C, Supplementary Fig. 3B, C, Table 1). Furthermore, in these lymph nodes, single cell death of lymphocytes in the paracortex and the medullary cord and neutrophil infiltration in the medullary sinus were observed on day 5 (Fig. 4B, C, Supplementary Fig. 3B, C, Table 1). In the thymus, single cell death of lymphocyte was observed in the cortex from day 3, and it was more pronounced on day 5 (Fig. 4D, Supplementary Fig. 3D, Table 1). In LM, these findings were not observed in the spleen and lymph nodes on day 7 (Supplementary Fig. 3, Table 1).

### Daily changes in serum biochemical parameters and cytokine/chemokine levels

**Fig. 5:**
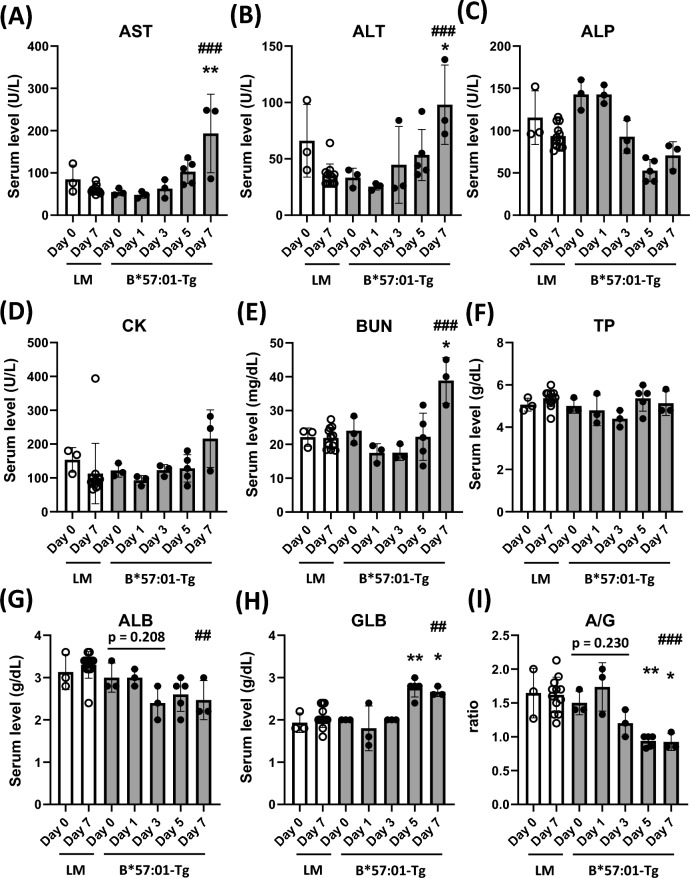
Daily changes in serum biochemical parameters. The serum levels including aspartate aminotransferase (AST; **a**), alanine aminotransferase (ALT; **b**), alkaline phosphatase (ALP; **c**), creatine kinase (CK; **d**), blood urea nitrogen (BUN; **e**), total protein (TP; **f**), albumin (ALB; **g**), globulin (GLB; **h**), albumin/globulin ratio (A/G; **i**). Data are expressed as the mean ± S.D. (n = 3–5/group). There were significant differences (*p < 0.05, **p < 0.01) compared with day 0 (one-way ANOVA, followed by Dunnett’s multiple comparisons tests), and significant differences (^##^*p* < 0.01, ^###^*p* < 0.001) between LM and B*57:01-Tg on day 7 (*t*-tests)

Changes in blood biochemical parameters, which can fluctuate with organ damage, were evaluated by measurement at 0, 1, 3, 5, and 7 days after ABC administration in B*57:01-Tg and LM. In B*57:01-Tg, AST level was exceeded 100 U/L in 3/5 of the individuals on day 5 and significantly increased on day 7, and ALT was significantly increased on day 7 (Fig. 5A, B). Although ALP and CK were also measured as deviation enzymes, no significant increases were observed (Fig. 5C, D). Significant increase in BUN was noted on day 7 in B*57:01-Tg (Fig. 5E). No significant difference was observed in TP (Fig. 5F). ALB level decreased after day 3, however, the differences were not statistically significant, and GLB increased significantly after day 5, and consequently, a significant decrease in A/G was observed after day 5 in B*57:01-Tg (Fig. 5G–I). No such changes were observed in LM (Fig. 5).

**Fig. 6:**
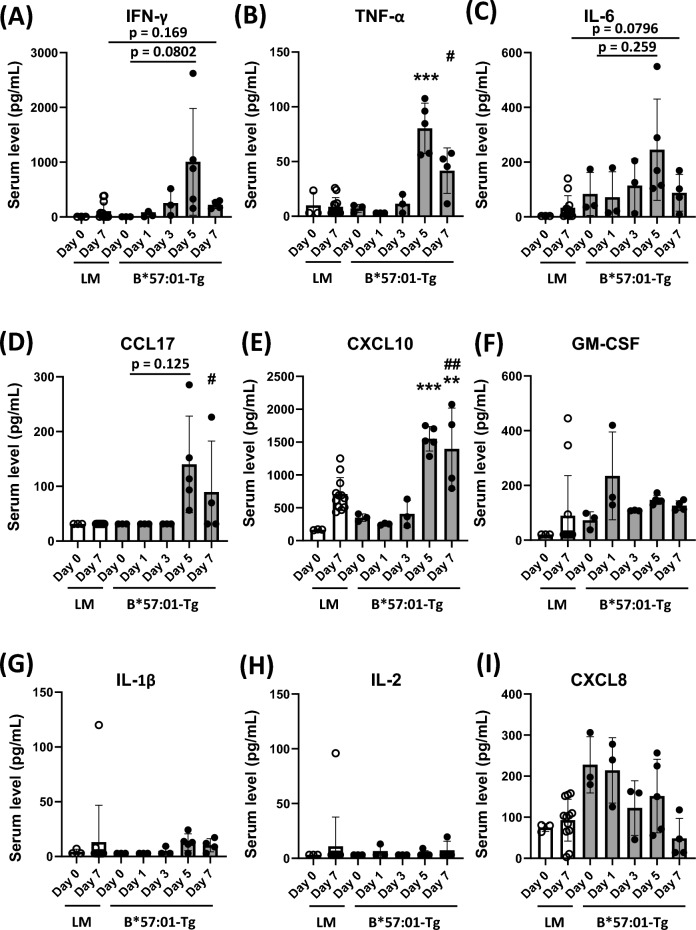
Daily changes in serum cytokine/chemokine levels. Serum levels of IFN-γ (**a**), TNF-α (**b**), IL-6 (**c**), CCL17 (**d**), CXCL10 (**e**), GM-CSF (**f**), IL-1β (**g**), IL-2 (**h**), and CXCL8 (**i**) in B*57:01-Tg with oral administration of abacavir. Below the limit of detection is plotted as 2.94 (**a**), 3.04 (**b**), 31.25 (**d**), 2.99 (**g**), or 3.22 (**h**) pg/mL. Data are expressed as the mean ± S.D. (n = 3–5/group). There were differences (**p < 0.01, ***p < 0.001) compared with day 0 (one-way ANOVA, followed by Dunnett’s multiple comparisons tests), and differences (^#^*p* < 0.05, ^##^*p* < 0.01) between LM and B*57:01-Tg in day 7 (*t*-tests)

Changes in blood biomarkers associated with the development and progression of systemic inflammation were evaluated by daily measurements of serum cytokine and chemokine levels. ABC was administered daily to B*57:01-Tg and LM, and serum cytokine and chemokine levels were measured on days 0, 1, 3, 5, and 7. In B*57:01-Tg, serum levels of tumor necrosis factor (TNF)-α and C-X-C motif chemokine ligand (CXCL) 10 increased significantly, and serum levels of interferon (IFN)-γ, interleukin (IL)-6, and CCL17 increased on day 5 but the differences were not statistically significant (Fig. 6A–E). Conversely, no considerable changes were observed in serum levels of granulocyte–macrophage colony-stimulating factor (GM-CSF), IL-1β, IL-2, and CXCL8 (Fig. 6F–I). In LM, on day 7, no significant increase in these serum levels was observed, or the values were significantly lower than B*57:01-Tg (Fig. 6).

## Discussion

Clinically, patients with cutaneous adverse drug reactions induced by ABC commonly develop systemic symptoms, such as fever and general malaise [3, 4]. Although the pathological changes in the skin of patients with drug eruptions are well-characterized, the details of what occurs in the body during the development of drug eruptions remain unclear [18]. This study showed that weight loss and thymic atrophy preceded skin eruption in ABC-administered B*57:01-Tg, but not in LM. These results suggest that some HLA genotype-dependent stress is induced by medications even before the onset of skin eruptions. In addition, inflammatory cell infiltration occurred in various organs simultaneously with skin eruption in ABC-administered B*57:01-Tg, suggesting that HLA-mediated ABC-induced effects occurred systemically.

We have previously reproduced abacavir-induced skin rash using B*57:01-Tg and obtained findings regarding cutaneous manifestations such as rash, infiltration of CD8^+^ T cells into skin tissues, and elevated serum CCL17 levels[15, 16]. In those experiments, we also encountered a situation in which many mice died during ABC dosing. The infiltration of inflammatory cells into skin tissues was so mild that it was unlikely that the mice died from skin inflammation, thus, we wondered if there was also an HLA-B*57:01-dependent effect of abacavir on tissues other than skin. Therefore, in this study, we expected that the pathological analysis of various organs of this mice model yield interesting insights into understanding the pathogenesis of deadly drug eruptions. We attempted to analyze the pathology of various organs and observed the following sequential changes. On day 1, weight loss was observed, suggesting that a stress response may have occurred immediately after administration (Fig. 1D). On day 3, thymic atrophy occurred, and increase in serum IFN-γ and decrease in serum ALB were recorded but the differences were not statistically significant, suggesting a systemic effect (Figs. 4D, 5G, 6A). On day 5, body temperature rose and skin eruption occurred (Fig. 1B, E). Furthermore, on day 5, inflammatory cell infiltration was observed in the skin, liver, kidney, and lungs, and increases in serum levels of GLB and various cytokines and chemokines were also observed, indicating systemic inflammation (Figs. 2, 3, 5H, 6A–E). On day 7, the AST level was markedly elevated, and a slight increase in ALT was also observed, suggesting that cellular injury may have occurred in various tissues, including the liver, in association with inflammatory cell infiltration (Fig. 5A, B). The ALT level was elevated by ABC administration while the ALP level was not (Fig. 5B, C), suggesting hepatocellular injury rather than cholestasis in the liver. The serum level of CK, a myocardial deviation enzyme, was not increased and no finding related with ABC administration was observed in the heart in the histopathological examination (Fig. 5D, Table 1), suggesting no significant myocardial damage. Even though serum BUN levels were elevated on day 7, the histopathological examination of the kidneys showed no obvious damage, suggesting an extrarenal regulation of BUN level (Fig. 5E).

B*57:01-Tg, but not LM, showed body weight loss and thymic atrophy. Notably, these were observed immediately after the administration of ABC (Figs. 1D, 4D). Clinical case reports of AHS patients indicated that some experienced symptoms, such as myalgia, before the onset of skin eruptions [6]. Taken together, these clinical case reports and our observations in mice suggest that HLA genotype-dependent systemic signs may precede the appearance of skin eruptions in the pathogenesis of AHS. HLA genotype-dependent drug hypersensitivity is considered to be caused by acquired immunity, which requires a specific timeframe [19]. In addition to antigen presentation by HLA, various co-stimulatory factors, such as activation of innate immunity, signals from barriers including the epidermis, and stress responses, are considered necessary to induce acquired immunity [20–22]; HLA genotypes are thought to have little effect on these factors. Therefore, an HLA genotype-dependent response immediately after ABC administration was unexpected. Although the detailed mechanism underlying this phenomenon is currently unknown, previous studies have suggested that innate immunity and stress response activation are involved in the pathogenesis of AHS. Martin et al. [23] showed that HSP70-induced innate immunity may be involved and that ABC induces HSP70 expression in the endoplasmic reticulum of PBMCs from patients with AHS. As HSP70 is induced by an unfolded protein response [24], HLA-B*57:01 may form abnormal HLA complexes upon exposure to ABC, causing an unfolded protein response. Shirayanagi et al. [25] reported an increased formation of aberrant HLA complexes in HLA-B*57:01-expressing cells upon exposure to ABC. Therefore, we hypothesized that the aberrant HLA complex induces HSP70 by triggering an unfolded protein response and that the downstream immune response is involved in the early pathogenic process of AHS. However, further studies are needed to confirm this hypothesis.

Biomarkers that predict the occurrence of idiosyncratic adverse drug reactions, such as drug eruptions, including AHS, have not yet been established, making it challenging to avoid side effects [18]. Idiosyncratic adverse drug reactions are exceptionally rare, and comprehensive examinations are seldom conducted before significant symptoms arise [26]. Animal models can be valuable in identifying biomarkers that can effectively predict the occurrence of drug eruptions. The mice treated daily with ABC showed elevated serum IFN-γ levels and decrease serum ALB levels in some individuals before the onset of skin eruptions and inflammatory cell infiltration into multiple organs (Figs. 1B, 2, 3, 4, 5G, 6A). These findings again indicate that there can be signs of systemic inflammation during the process that leads to AHS. The increased IFN-γ may have induced subsequent increases in CCL17 and CXCL10 levels [27, 28], which may have been involved in the induction of skin inflammation. Gao et al. [16] reported that CCL17 is involved in T-cell infiltration into the skin. Consistent with the previous observation, elevated serum CCL17 levels were observed after day 5 (Fig. 6D), which coincided with skin eruption and CD8^+^ T cell infiltration (Figs. 1B, 2B). The involvement of CCL17 in the infiltration of CD8^+^ T cells into other tissues is unlikely [16], and further studies are required to clarify the underlying mechanism. Notably, on day 5 (Fig. 6), some individual mice exhibited markedly elevated levels of IFN-γ, TNF-α, and IL-6, although these levels appeared to decrease on day 7. Tracking the same individuals throughout the study period was not feasible as blood and organs were collected from each mouse at each time point. This implies that mice with markedly elevated cytokines on day 5 might have subsequently progressed to a cytokine storm-like syndrome [29] and died before day 7. However, due to the unavailability of data immediately before death, establishing a definitive relationship between the cause of death and cytokine levels was challenging. At present, the current study has not identified any biomarkers that predict the onset of skin eruption, i.e., biomarkers prior to day 5, and the relationship between cause of death and cytokines. A more comprehensive analysis that follows the same individual mouse from before the onset of hypersensitivity to just before death is needed.

In our mouse model, the fatality rate on day 7 was 70%, which is considerably higher than what is observed in clinical practice [3, 4]. This may be due to the loading of extreme conditions that readily induced immune activation, such as PD-1 knockout and CD4^+^ T-cell depletion. The infiltration of inflammatory cells into each organ, including the skin, was very mild, making it unlikely that the mice died from inflammation in the skin, liver, kidneys, or lungs. Systemic inflammation leading to death is believed to be the result of the cumulative inflammatory and immune responses in multiple organs. In fact, patients with AHS often exhibit elevated levels of serum markers associated with liver or kidney injury, including AST, ALT, ALP, lactate dehydrogenase, and creatinine [4, 6, 30, 31]. Therefore, patients with AHS may have lesions in several tissues, such as liver and kidneys, even if they do not show any noticeable symptoms. Moreover, some patients go into shock and die; however, they rarely show significant damage to specific organs [4, 31, 32]. While direct extrapolation of the results to clinical practice is challenging owing to variations in specific backgrounds of the mice, the findings imply that when administering ABC to HLA-B*57:01 carriers, it is crucial to consider early systemic inflammation and its impact on organs beyond the skin.

In conclusion, the histopathological examination of a mouse model of ABC-induced skin eruption shows that disorders in various organs other than the skin should be considered and provides insights into the unexpected early systemic responses dependent on HLA-B*57:01.

### Supplementary Information

Below is the link to the electronic supplementary material.

Supplementary Fig. 1 Histopathological examination of auricular skin in B*57:01-Tg and LM (PDF 493 KB)

Supplementary Fig. 2 Histopathological examination of liver, kidney, and lung in B*57:01-Tg and LM (PDF 642 KB)

Supplementary Fig. 3 Histopathological examination of lymphoid tissues in B*57:01-Tg and LM (PDF 1067 KB)

## Data Availability

The data could be obtained upon reasonable request to the corresponding authors.
