# Supplementary material for: Pathological changes in various organs in HLA-B*57:01 transgenic mice with abacavir-induced skin eruption
Source: Toxicol Res. 2024 Jan 6;40(2):223–35. doi: 10.1007/s43188-023-00220-1 (PMC10959918; doi:10.1007/s43188-023-00220-1)
Supplement: Supplementary file 2 — Supplementary Fig. 2 Histopathological examination of liver, kidney, and lung in B*57:01-Tg and LM (PDF 642 KB) [file 43188_2023_220_MOESM2_ESM.pdf]

Supplementary Fig. 2

Pathological changes in various organs in HLA-B*57:01 transgenic mice with abacavir-induced skin eruption

Akira Kazaoka[1], Kazuyoshi Kumagai[2], Junya Matsushita[2], Tetsuo Aida[2], Saki Kuwahara[1], Shigeki Aoki[1]*, Kousei Ito[1]*

[1]Laboratory of Biopharmaceutics, Graduate School of Pharmaceutical Sciences, Chiba University, Chiba, Japan.

[2]Medicinal Safety Research Laboratories, Daiichi Sankyo Co., Ltd, Tokyo, Japan.

***Corresponding authors:**

Shigeki Aoki, Ph.D.

Laboratory of Biopharmaceutics, Graduate School of Pharmaceutical Sciences, Chiba University, 1-8-1 Inohana, Chuo-ku, Chiba-city, Chiba 260-8675, Japan.

Tel: +81 43 226 2888, Fax: +81 43 226 2888, E-mail: aokishigeki@chiba-u.jp

Kousei Ito, Ph.D.

Laboratory of Biopharmaceutics, Graduate School of Pharmaceutical Sciences, Chiba University, 1-8-1 Inohana, Chuo-ku, Chiba-city, Chiba 260-8675, Japan.

Tel: +81 43 226 2886, Fax: +81 43 226 2886, E-mail: itokousei@chiba-u.jp

Supplementary Fig. 2

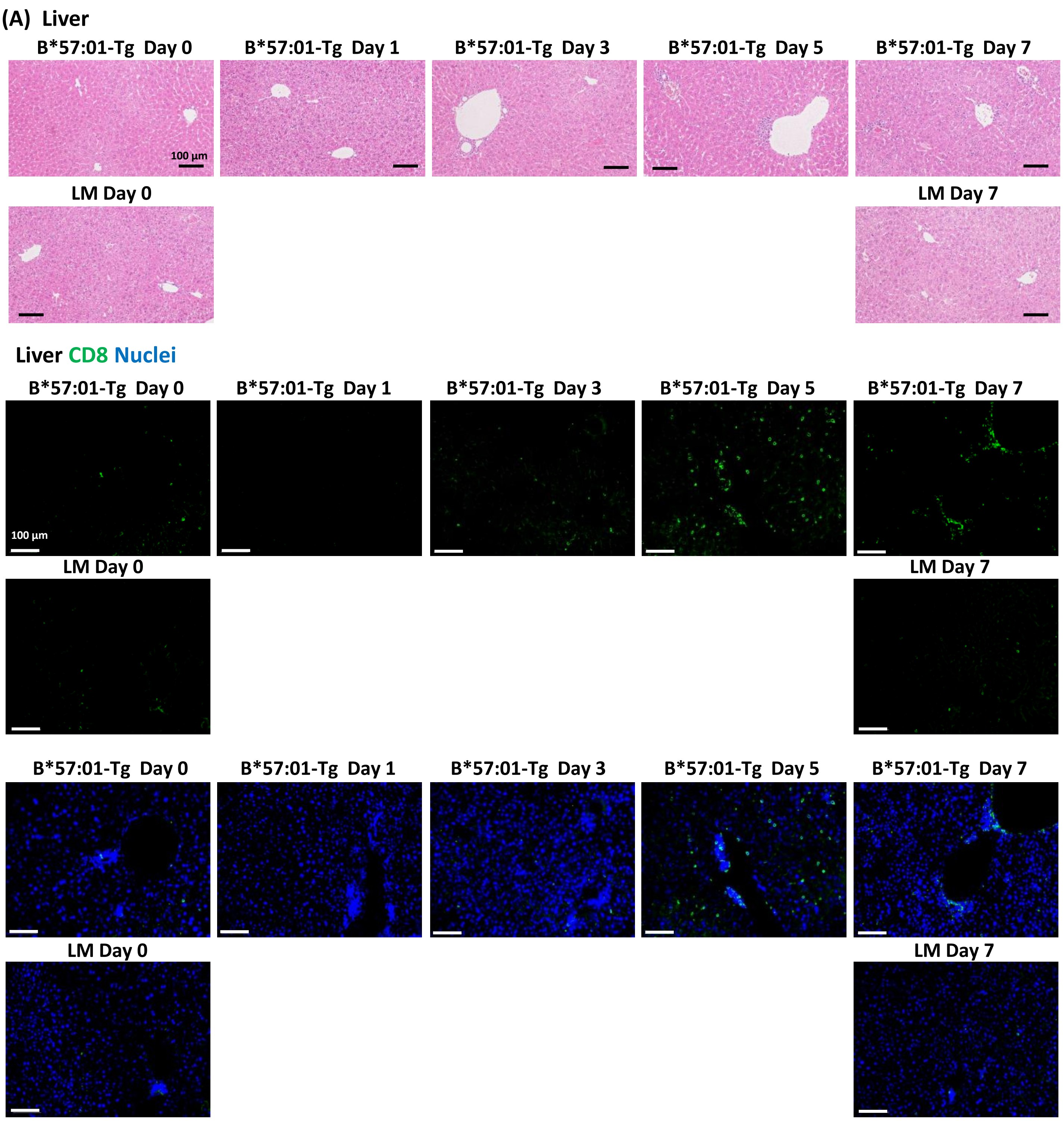

Supplementary Fig. 2 (Continued)

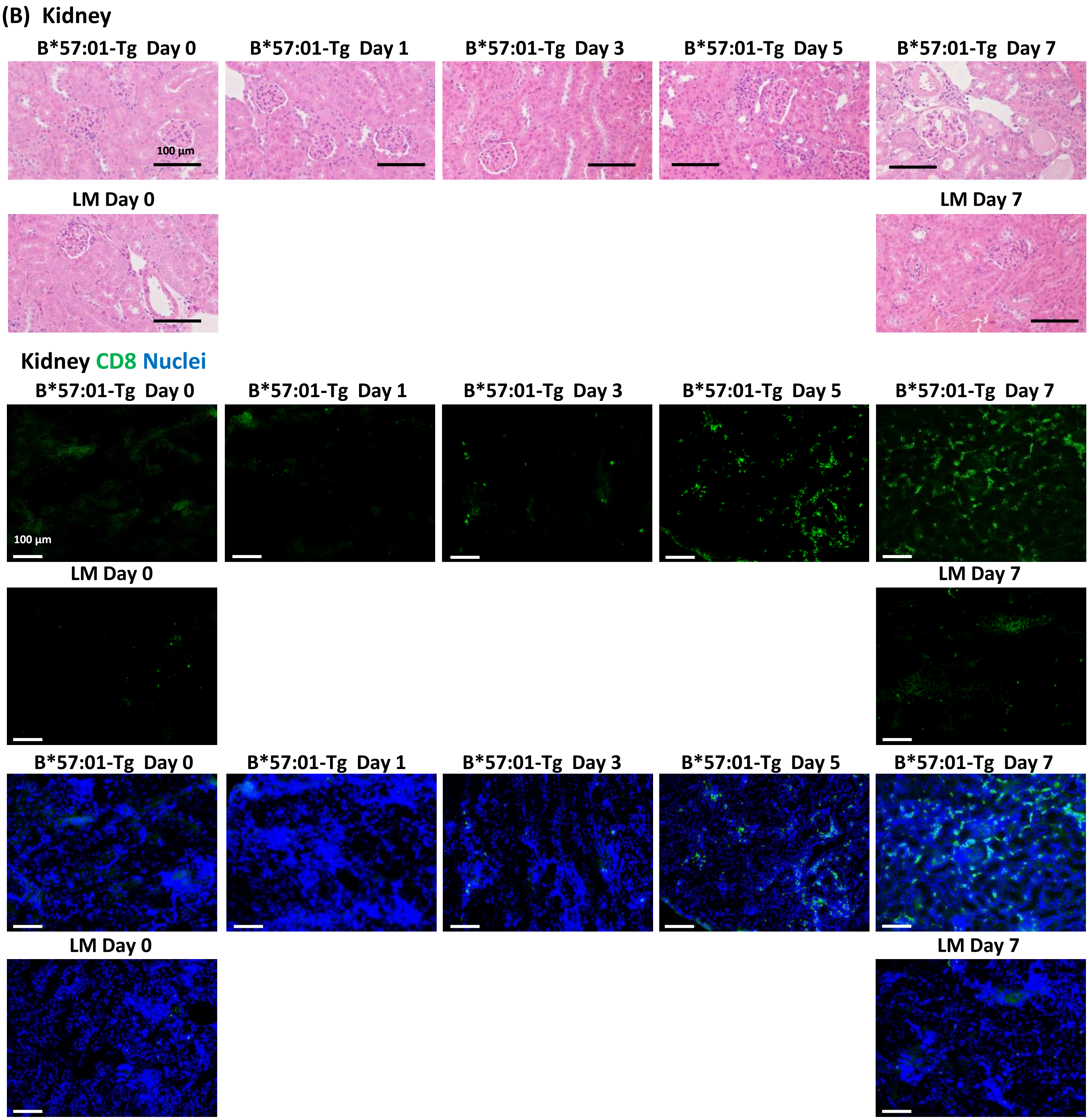

Supplementary Fig. 2 (Continued)

(C) Lung

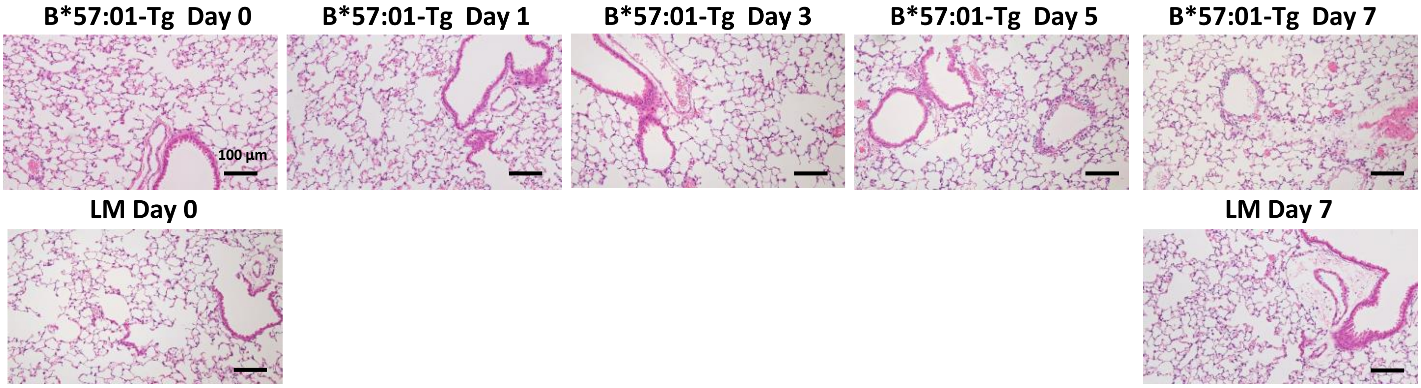

Supplementary Fig. 2 Histopathological examination of liver, kidney, and lung in B*57:01-Tg and LM

Representative images of the sections of liver (A), kidney (B), and lung (C) stained with hematoxylin and eosin and with anti-CD8 antibody (green) and Hoechst 33342 (blue; nuclear staining) in B*57:01-Tg and LM with oral administration of abacavir.
